# Multiple High-Affinity K^+^ Transporters and ABC Transporters Involved in K^+^ Uptake/Transport in the Potassium-Hyperaccumulator Plant *Phytolacca acinosa* Roxb

**DOI:** 10.3390/plants9040470

**Published:** 2020-04-08

**Authors:** Qin Xie, Liying Ma, Peng Tan, Wentao Deng, Chao Huang, Deming Liu, Wanhuang Lin, Yi Su

**Affiliations:** 1 Hunan Provincial Key Laboratory of Phytohormones and Growth Development, Hunan Agricultural University, Changsha 410128, China; xieqin@stu.hunau.edu.cn (Q.X.); liudeming825@hunau.edu.cn (D.L.); 2 College of Bioscience and Biotechnology, Hunan Agricultural University, Changsha 410128, China; liyingma@stu.hunau.edu.cn (L.M.); tanpeng@novogene.com (P.T.); xppyyfd@163.com (W.D.); chaoh@hunau.edu.cn (C.H.)

**Keywords:** *Phytolacca acinosa*, potassium transport, high-affinity K^+^ transporters, ABC transporters

## Abstract

Potassium is an important essential element for plant growth and development. Long-term potassium deprivation can lead to a severe deficiency phenotype in plants. Interestingly, *Phytolacca acinosa* is a plant with an unusually high potassium content and can grow well and complete its lifecycle even in severely potassium deficient soil. In this study, we found that its stems and leaves were the main tissues for high potassium accumulation, and *P. acinosa* showed a strong ability of K^+^ absorption in roots and a large capability of potassium accumulation in shoots. Analysis of plant growth and physiological characteristics indicated that *P. acinosa* had an adaptability in a wide range of external potassium levels. To reveal the mechanism of K^+^ uptake and transport in the potassium-hyperaccumulator plant *P. acinosa*, K^+^ uptake-/transport-related genes were screened by transcriptome sequencing, and their expression profiles were compared between K^+^ starved plants and normal cultured plants. Eighteen members of HAK/KT/KUPs, ten members of AKTs, and one member of HKT were identified in *P. acinosa*. Among them, six *HAKs*, and two *AKTs* and *PaHKT1* showed significantly different expression. These transporters might be coordinatively involved in K^+^ uptake/transport in *P. acinosa* and lead to high potassium accumulation in plant tissues. In addition, significantly changed expression of some ABC transporters indicated that ABC transporters might be important for K^+^ uptake and transport in *P. acinosa* under low K^+^ concentrations.

## 1. Introduction

Potassium (K) is an important nutrient element in plant growth and development. Cellular K^+^ is involved in multiple physiological processes, e.g., ion homeostasis, stress response, membrane potential formation, guard cell movement, enzyme activity maintenance, cell expansion, and photosynthesis in plants [1,2,3,4,5]. Plants are sensitive to changes in the concentration of K^+^ in the external environment during growth and development. Plants show growth and development defects under long-term low K^+^ stress [6]. Therefore, maintaining a certain level of cellular K^+^ is very important for plant growth and development. Cytosolic potassium has been directly measured in plants, and values higher than 100 mM are uncommon in terrestrial plants [3,7,8,9]. For supplying the normal demand for K^+^ concentration in vivo, plants intake K^+^ mainly through the root to maintain the cellular K^+^ homeostasis and general bioactivity of multiple molecules [10,11].

Intracellular K^+^ is located in cytoplasm and organelles such as vacuoles, endo-plasmic reticulum, mitochondria, and chloroplasts. For optimal performance, K^+^ concentrations in plant cell must be maintained at about 100 to 150 mM [3], and thus plants evolve multiple K^+^ transport systems across a membrane. K^+^ uptake by root cells from soil solution is a highly efficient process and not usually limiting to K^+^ uptake. It initially hypothesizes that K^+^ influx to root cells is mediated by distinct “high-affinity” and “low-affinity” transporters, operating at low (<1 mM) and high (>1 mM) rhizosphere K^+^ concentrations, respectively [12]. The increasing expression of genes encoding high-affinity systems play an important role in K^+^ influx when K^+^ is in short supply [13]. In addition, K^+^ uptake driven by thermodynamics is also a dominant manner besides the reason of “affinity” for K^+^. The pH and voltage gradients generated by H^+^-ATPase supply the driving energy for K^+^ influx across the plasma membrane of root cells in apoplasmic solution containing less than 1 mM K^+^ [8,14]. H^+^/K^+^ symporters, such as NHXs and CHXs, mediate this process and are vital important in K^+^ homeostasis in vacuolate plant cells [15,16,17,18]. At rhizosphere K^+^ concentrations above 1 mM, K^+^ influx to root cells facilitated by K^+^ channels is the dominant mode because K^+^ tends to be thermodynamic equilibrium across the tonoplast in K^+^-starved roots [8,14]. For example, *Arabidopsis* Potassium Channel 1 (AtKC1) modulates root hair K^+^ influx in *Arabidopsis thaliana* [19]. In K^+^-replete root, K^+^/H^+^-antiporters can actively transport K^+^ into the vacuoles to stabilize cytoplasmic K^+^ level [20,21]. 

K^+^ influx to plant cells can be mediated by cation/H^+^ symporters, K^+^/Na^+^ symporters, and/or K^+^-permeable cation channels depending upon the K^+^ electrochemical gradient. Its efflux from plant cells occurs through outward rectified K^+^ channels (KORCs) and non-specific outward-rectifying cation channels (NORCs) [1,21]. K^+^ across vacuoles membrane are mediated by cation/H^+^ antiporters, vacuolar K^+^ (VK) channels, fast-activating vacuolar (FV) channels, and slowly activating vacuolar (SV) channels. KUP/KT/HAKs belong to the high-affinity K^+^ transport system. The expression of KUP/KT/HAK family genes is affected by external K^+^ concentrations, and many of them are usually upregulated under low K^+^ levels [22]. In *Arabidopsis*, AtHAK5, AtKUP1, AtKUP2, AtKUP11, and AtCHX13 have been found in plasma membranes of various cell types and are thought to catalyze K^+^ influx to cells at low apoplastic K^+^ concentrations [16,23,24,25]. *OsHAK1* and *OsHAK5* in rice (*Oryza sativa*) contribute to K^+^ absorption in plants under low K^+^ levels [26,27,28]. In the *OsHAK1* knockout transgenic line, the extension of rice root cells was inhibited, and the net absorption rate of K^+^ was decreased [27]. *OsHAK5* is mainly expressed in the root surface, pericycle, and vascular tissue [28]. *OsHAK10*, located on the vacuole membrane, can promote the outflow of K^+^ in the vacuole under conditions of K^+^ deficiency and thus maintains the balance of K^+^ in cells [29]. In addition, K^+^/Na^+^ cotransporters encoded by members of the *HKT/Trk* gene family are also found in the plasma membranes of plant cells [24]. Plants have also evolved some nonspecific transporters which mediate the uptake and transport of multiple ions/molecules [30].

Total potassium in soil is the sum of water-soluble potassium, exchangeable potassium, non-exchangeable potassium, and structured potassium. Among them, water-soluble potassium and exchangeable potassium supply the available potassium, which supports plant growth and development and is one of the most important indicators reflecting the degree of soil fertility. In most cases, higher plants absorb K^+^ mainly through the high-affinity system because the concentrations of available potassium in soils are lower than those of cellular K^+^. The soil potassium content is not uniform in different type of soils, and potassium is unevenly distributed in different regions. In China, 23% of cultivated land (more than 23 million ha) shows K^+^ deficiency (available potassium < 70 mg/kg [31]). In South China, available potassium in most farmlands cannot support normal growth and development of multiple crops, and thus, millions of tons of potassium fertilizer need to be imported from other countries. Although much of the land is relatively deficient in potassium, we still screened some low-potassium-tolerant plants, e.g., *Phytolacca acinosa*, *Alternanthera philoxeroides*, and *Celosia argentea*. Among them, *P. acinosa* shows a strong ability to adapt to a low K^+^ environment and has a high capability for potassium accumulation in its aerial parts. Thus, *P. acinosa* is an ideal material for use as a biological potassium fertilizer. In our previous research, *PaHAK1* identified in *P. acinosa* was proven to be important for K^+^ uptake in roots under a low potassium environment [32]. However, the mechanism of K^+^ transportation and accumulation are not clear in the potassium-hyperaccumulator plant *P. acinosa*. In this study, we collected multiple samples from different regions in Hunan Province and analyzed the differences of potassium content in *P. acinosa* tissues. The collected *P. acinosa* was characterized by high potassium and adaptability in a wide range of external K^+^ levels. Using a global transcriptomic analysis method, we identified some candidate transporters contributing to K^+^ transportation and accumulation. We hypothesized that multiple high-affinity transporters cooperatively regulate K^+^ transportation and accumulation and, moreover, that ABC transporters play important roles in these processes.

## 2. Results

### 2.1. P. acinosa Showed High Potassium Characteristics

**Figure 1:**
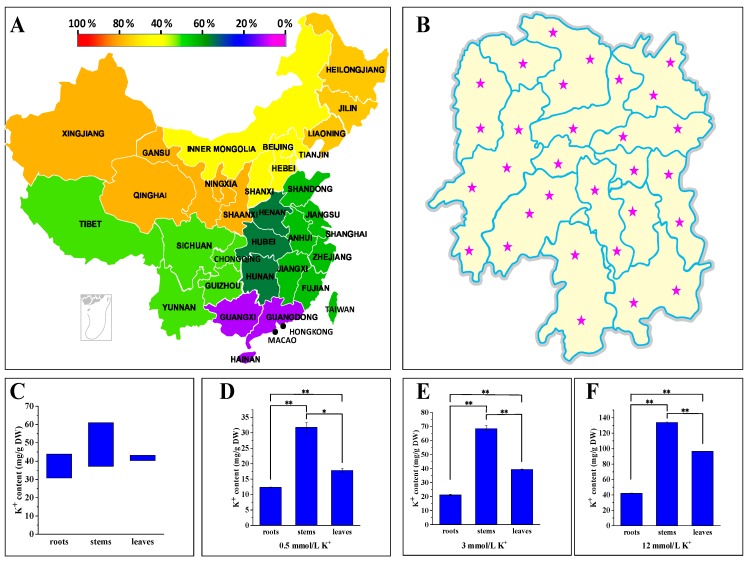
(**A**) The area percentage of available potassium (> 100 mg/ kg) in fields in the main regions of China; (**B**) Sample collection sites in Hunan Province; (**C**) Content ranges of potassium in different tissues of *Phytolacca acinosa* collected from fields; (**D**–**F**) Potassium accumulation in different tissues of *P. acinosa* under different K^+^ treatments. Data shown as means ± SD of three biological replicates (*n* = 30). Asterisks indicate a significant difference based on a Dunnett’s test. * significant difference at 5% level (*P* < 0.05); ** significant difference at 1% level (*P* < 0.01).

Total potassium content in soil varies enormously in different types of soil and in different areas of China [33]. As the direct utilizable potassium for plants, available potassium also has an extremely unbalanced distribution in soil. Altogether, the soils in Northwest China, Northeast China, and North China are rich in potassium, but in Central China, South China, and East China, the contents of available potassium in fields are generally lower than 100 mg/kg (Figure 1A). Especially in South China, available potassium in most farmlands cannot meet the needs of crop growth and development, and thus, millions of tons of potassium fertilizer need to be imported from other countries according to the data from National Bureau of Statistics in 2019. According to the Standard for Nutrient Classification of Soil Census in China [31], Hunan Province is located in the south of Central China, and its farmland is typically lacking in potassium. The content of available soil potassium is lower than 100 mg/kg in about 80% of the areas, and half of the lands show severe potassium deficiency (available potassium < 50 mg/kg [31]) in Hunan Province. We carried out a field plant survey and found that *P. acinosa* is widely distributed in various types of soil in Hunan. *P. acinosa* is an annual herb, and dormant buds in its root tubers can develop into new shoots in the following year. The mass of *P. acinosa* tuberous roots can increase year by year, and their weight can even reach several kilograms. Along with the increase of tuberous root mass, aerial parts of the biomass also increase year after year, and the height of *P. acinosa* can reach more than 2 m. We collected multiple samples of *P. acinosa* from different regions of Hunan to reveal the characteristics of potassium accumulation (Figure 1B). Potassium accumulation in the stems was significantly higher than that in the roots and leaves (Figure 1C). In the field, potassium accumulation in the stems was in a relatively large range (37.17–60.86 mg/g dry weight), but the potassium content in leaves showed little change (ranging from 40.33 to 43.28 mg/g dry weight). Thus, stems and leaves are the main tissues for high potassium accumulation in *P. acinosa*. Generally, the potassium contents often range from 2% to 10% dry weight in plants [3,21]. The critical K^+^ concentration in plant tissue is generally lower than 3% (DW) [3,32,34,35]. In K^+^-sufficient plants, these concentrations can be up to approximately 10% (DW), exceeding those required to support near-maximal rates of growth [3,36]. Moreover, Hu (1980) did a field survey to screen high K^+^ plants in Hunan Province of China but found that plant species with a K^+^ content more than 3% (DW) were very few (Supplementary Material). These naturally growing plants with more than 3% can be regarded as potassium-hyperaccumulator plants [34]. The results showed that *P. acinosa* was a potassium-hyperaccumulator plant.

Through the field plant survey, we found that the content of potassium ion varies along with soil type. To further clarify the relationship between potassium accumulation in *P. acinosa* tissues and available potassium concentration in the rhizosphere, we cultured seedlings of *P. acinosa* under various K^+^ concentrations and analyzed the potassium contents in different tissues. The results showed that the potassium content in *P. acinosa* increased along with the external K^+^ content. Potassium accumulation in stems was also significantly higher than that in leaves and roots under different treatments (Figure 1D–F). The translocation coefficients under 0.5, 3.0, and 12 mmol/L K^+^ treatments were 4.01, 5.08, and 5.48, respectively. *P. acinosa* had a high translocation coefficient under lower K^+^ concentrations, which indicated that *P. acinosa* has a strong ability for K^+^ absorption in roots and a large capability of potassium accumulation in shoots.

### 2.2. P. acinosa Shows Adaptability to a Wide Range of External Potassium Levels

**Figure 2:**
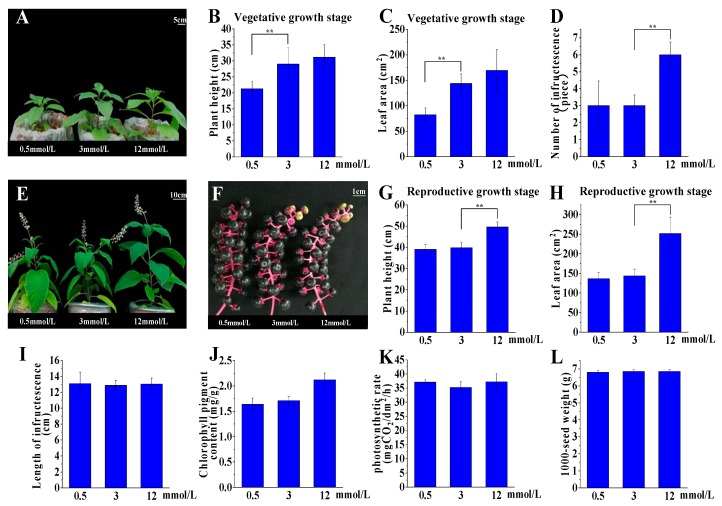
(**A**) The growth status of *P. acinosa* under different potassium treatments at vegetative growth stage. (**B**) Plant height of *P. acinosa* under different potassium treatments at vegetative growth stage. (**C**) Leaf area at vegetative growth stage under different potassium treatments. (**D**) Number of infructescences under different potassium treatments. (**E**) The growth status of *P. acinosa* under different potassium treatments at reproductive growth stage. (**F**) Growth status of infructescences under different potassium treatments. (**G**) Plant height of *P. acinosa* under different potassium treatments at reproductive growth stage. (H) Leaf area at reproductive growth stage under different potassium treatments. (**I**) Length of infructescences under different potassium treatments. (**J**) Chloroplast content under different potassium treatments. (**K**) Effect of potassium treatments on photosynthesis of *P. acinosa*. (**L**) The 1000-seed weight of *P. acinosa* under different potassium treatments. Data shown as means ± SD of three biological replicates (*n* = 30). Asterisks indicate a significant difference based on a Dunnett’s test. * significant difference at 5% level (*P* < 0.05); ** significant difference at 1% level (*P* < 0.01).

We analyzed the growth and development of *P. acinosa* collected from fields and found that *P. acinosa* could grow well and complete its lifecycle even in severely potassium deficient soil (available potassium < 50 mg/kg [31]). Furthermore, growth occurred in three conditions: K^+^ deficiency (0.5 mmol/L), K^+^ average (3.0 mmol/L), and K^+^ excess (12 mmol/L). At the vegetative growth stage, we found that the plant height and leaf area of *P. acinosa* increased along with the increase in K^+^ concentration (Figure 2A–C). The number of infructescences per plant was about 6 under 12 mmol/L K^+^ treatment, which decreased to about 3 under 0.5 and 3.0 mmol/L K^+^ treatments (Figure 2D). At the reproductive growth stage, there were no significant differences in plant height and leaf area under 0.5 and 3.0 mmol/L K^+^ treatments (Figure 2E–H). Additionally, no significant difference in length of infructescences was detected under these three treatments (Figure 2I). Interestingly, berries of *P. acinosa* were full and ripened with different degrees of maturity under low K^+^ conditions, but the degree of maturity of berries was not consistent under higher K^+^ concentrations (Figure 2F). We also found that the chlorophyll content and net photosynthetic rate showed no significant difference in the three treatments (Figure 2J,K). The 1000-seed weights showed no significant differences under the three treatments (Figure 2L). 

Overall, *P. acinosa* under 0.5 mmol/L K^+^ treatment did not show potassium deficiency symptoms, although the plant was relatively smaller compared with those under the 3.0 and 12 mmol/L K^+^ treatments throughout the entire lifecycle. *P. acinosa* cultured in K^+^ deficiency conditions could complete its lifecycle normally, and its growth and development showed no significant differences compared to when cultured under average K^+^ conditions. Therefore, *P. acinosa* demonstrated adaptability to a wide range of external potassium levels.

### 2.3. Global Transcriptome Analysis Reveals the Involvement of Multiple Biological Processes during Potassium Accumulation

High potassium contents were detected in *P. acinosa* tissues, especially in shoots. *P. acinosa* can grow well and show high adaptability in low K^+^ conditions. Compared to some other plants, *P. acinosa* showed lower *K_m_* (34.14 μM) and higher *V_max_* (0.147 μmol/g.min) [37,38]. *P. acinosa* might have higher K^+^ absorption efficiency through a high-affinity K^+^ transport system. *P. acinosa* might develop a more efficient mechanism for K^+^ uptake and accumulation. In our previous studies, we isolated a member of the high-affinity K^+^ transporter PaHAK1 from *P. acinosa*. Overexpression of *PaHAK1* in rice and *Arabidopsis* promoted K^+^ uptake in the transgenic plants [32]. However, more details are still needed to explain why *P. acinosa* tissues have the characteristic of high potassium accumulation. To reveal the mechanism of K^+^ uptake and transport in the potassium-hyperaccumulator plant *P. acinosa*, the expression profiles of K^+^ uptake-/transport-related genes were analyzed at the level of the global transcriptome. 

**Figure 3:**
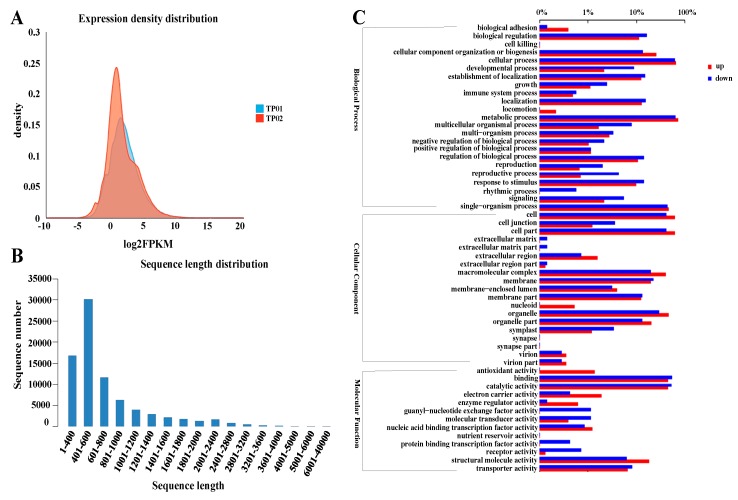
Global transcriptome analysis. (**A**) The density distribution map of gene expression. (**B**) The sequence length distribution of unigenes in *P. acinosa* under 0 and 3.0 mmol/L K^+^ treatment. (**C**) Classification of the genes in response to the K^+^ starvation treatment compared with the 3.0 mmol/L K^+^ treatment via Gene Ontology (GO) analysis.

RNA-Seq by Expectation-Maximization (RSEM) software was used to analyze the gene expression in samples of K^+^ starvation treatment and average K^+^ treatment (3.0 mmol/L). The samples under 3.0 mmol/L K^+^ treatment (average K^+^ treatment) were represented by TP01, and the samples under K^+^ starvation were represented by TP02. Basic data of RNA sequencing in the samples of TP01 and TP02 were displayed in Table 1. The Q20 ratio of TP01 ranged from 97.3% to 97.5%, and the Q20 ratio of TP02 ranged from 98.2% to 98.7%. The Q30 ratio ranged from 91.73% to 91.78% for TP01 and from 95.27% to 95.40% for TP02, respectively (Table 1). Furthermore, the expression of differentially expressed genes (DEGs, fold change (FC) ≥ 2 and *P*-value ≤ 0.05) between the two treatments were obtained, and gene expression was estimated using the fragments per kilobase per million (FPKM) method. The results indicate that the two samples had some non-overlapping expression patterns, and the gene expression density distributions were also different between the two samples (Figure 3A).

The total reads were assembled into 97,739 transcripts accounting for 79,768 unigenes using Trinity software. The N50s of unigenes and transcripts were 825 and 903 bp, respectively. For the unigenes, 21.01% had a length of 1–400 bp, 37.75% were 401–600 bp in length, and 0.01% were 6001–40,000 bp in length (Figure 3B). A total of 5886 unigenes were detected as DEGs in comparison to their FPKM values of TP01 and TP02. Among them, 3870 were upregulated, while 2016 were downregulated when comparing the K^+^ starvation treated sample (TP02) to the average K^+^ treated sample (TP01). The GO functional enrichment analysis showed that those DEGs included 55 subclasses and were involved in biological processes, cellular components, and molecular functions (Figure 3C).

### 2.4. Expression Profile of K^+^ Transporters Based on Transcriptome Sequence

**Figure 4:**
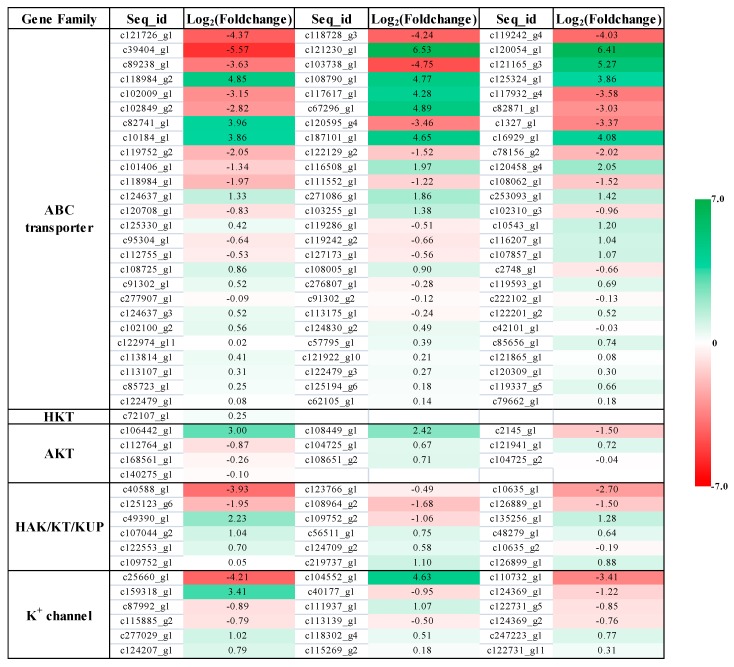
Expression profiles of transporter families. The green means upregulation and the red means downregulation of gene expression at K^+^ deficiency treatment. The value of 0 represents no change in gene expression between K^+^ starvation treatment (TP02) and normal K^+^ treatment (TP01). The value represents fragments per kilobase million (FPKM).

**Figure 5:**
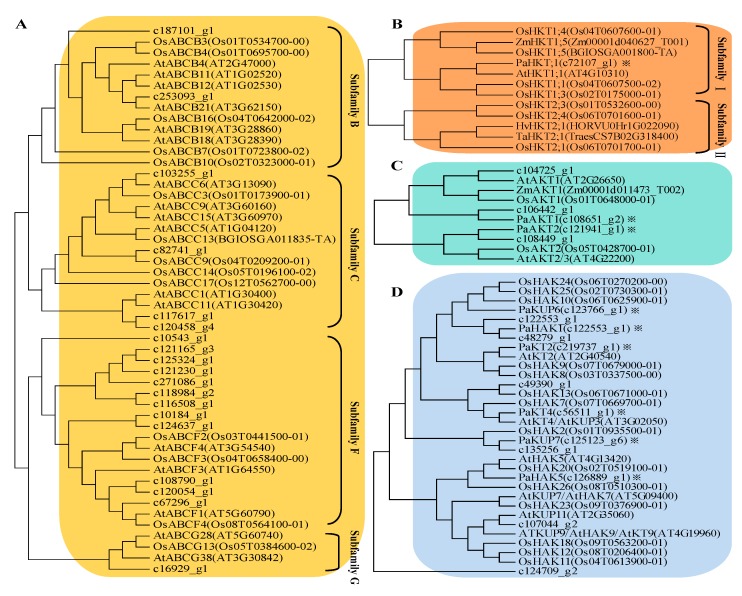
Phylogenetic analysis and nomenclature of target gene. (**A**) Phylogenetic analysis of ABC transporter family. (**B**) Phylogenetic analysis of HKT family. (**C**) Phylogenetic analysis of AKT family. (**D**) Phylogenetic analysis of HAK/KT/KUP family.

According to the transcriptional information, we found that significant changes took place for the expression of many ABC transporters in *P. acinosa* under the K^+^ starvation treatment compared to the average K^+^ treatment (Figure 4). ABC transporters belong to a superfamily, and more than 70 members were expressed in *P. acinosa*. The ABC transporter family has strong transport functions and can transport inorganic ions, amino acids, lipids, and metal ions [39]. In this study, the expression of 45 ABC transporters was upregulated and that of 33 members was downregulated under K^+^ starvation. Through phylogenetic analysis, we found that the upregulated genes in the ABC transporter family were mainly distributed in subfamilies of ABCB, ABCC, ABCF, and ABCG (Figure 5A). The ABC transporter may be important for K^+^ uptake and transport of *P. acinosa* under the conditions of K^+^ starvation. 

Specific K^+^ transporters mainly include three families: HAK/KT/KUP, AKT, and HKT. In this study, we screened 18 members of HAK/KT/KUPs, 10 members of AKTs, and 1 HKT member in *P. acinosa* by global transcriptome. Nine *HAK/KT/KUP* genes were upregulated, and nine *HAK/KT/KUP* genes were downregulated under the K^+^ starvation treatment. Five *AKT* genes were upregulated, and five *AKT* genes were downregulated. We also found six members of K^+^ channel under the condition of K^+^ starvation. Three members were upregulated, and two members were downregulated (Figure 4). Among them, the expression of six *HAK*s and two *AKTs* was significantly different in *P. acinosa* under the condition of K^+^ starvation. *HKT* gene expression was significantly upregulated. Through phylogenetic analysis, we found that these nine transporters were PaHAK1, PaHAK5, PaKT2, PaKT4, PaKUP6, PaKUP7, PaAKT1, PaAKT2, and PaHKT1, respectively (Figure 5B–D). 

### 2.5. Expression Pattern of Potassium Transport Genes in P. acinosa

**Figure 6:**
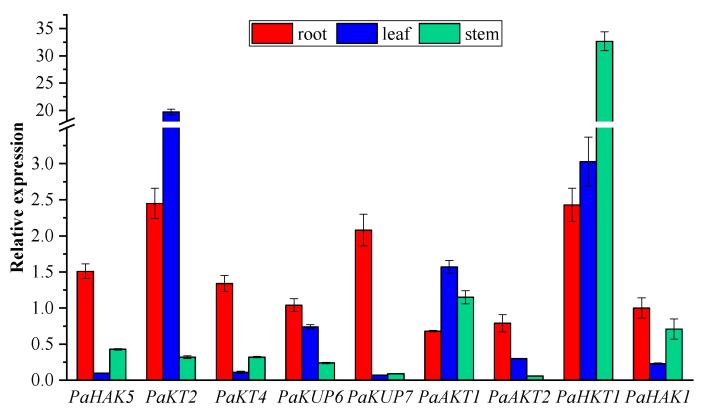
Expression level of high-affinity K^+^ transporter genes in different tissues. The expression of a known gene *PaHAK1* in root was normalized as value 1. Three biological replicates were performed in qRT-PCR (*n* = 3).

In our previous study, *PaHAK1* identified from *P. acinosa* was mainly expressed in roots and played an important role in K^+^ uptake [32]. In this study, we revealed the expression pattern of 9 K^+^ transporter genes in different tissues of *P. acinosa* at normal K^+^ levels through RT-qPCR. Genes with relatively high expression in roots included *PaHAK5*, *PaKT4*, *PaKUP6*, *PaKUP7*, and *PaAKT2* (Figure 6), which might mediate K^+^ uptake from soil. *PaKT2* and *PaKUP6* showed high expression in leaves (Figure 6). PaHAK5, PaKT4, PaKUP6, PaAKT1, and PaHKT1 might play important roles in the K^+^ transport in stems at normal K^+^ levels.

**Figure 7:**
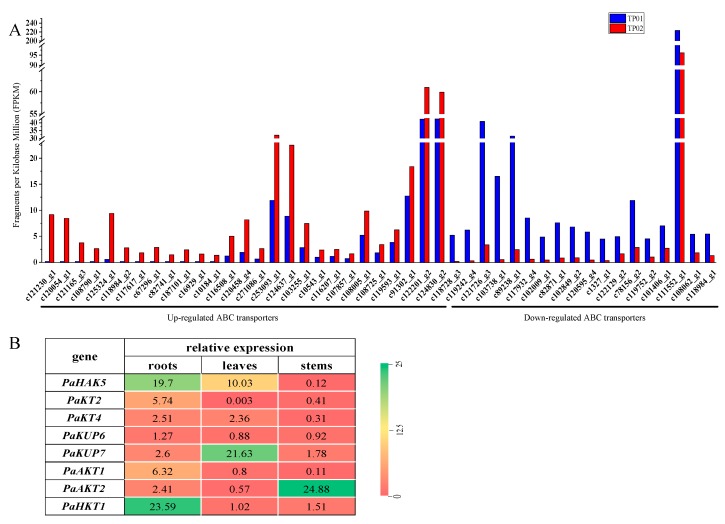
(**A**) Potassium starvation significantly regulated the expressions of ABC transporter genes. The values represent fragments per kilobase million (FPKM). The sample under 3.0 mmol/L K^+^ treatment (average K^+^ treatment) was represented by TP01, and the sample under K^+^ starvation was represented by TP02. (**B**) Low potassium induced the expression of high-affinity K^+^ transporter genes in different tissues. The value represents the ratio of gene expression in K^+^ deficiency vs. normal K^+^ level.

In addition, we analyzed the induced expression of K^+^ transporter genes under different K^+^ supply. Through transcriptome analysis, we found that the expression of 45 ABC transporters was upregulated and the expression of 33 members was downregulated under K^+^ starvation (Figure 4). Among them, 27 upregulated members and 18 downregulated members were significantly induced by K^+^ starvation (Figure 7A). These upregulated ABC transporters could be classed into subfamilies of ABCB, ABCC, ABCF, and ABCG, and might play important roles in nonspecific K^+^ uptake/transport in *P. acinosa*. Moreover, differential expression of specific K^+^ transporter genes in different tissues was compared between normal K^+^ (3.0 mmol/L) and K^+^ moderate deficiency (0.5 mmol/L) treatments through RT-qPCR. The multiple relationships between different K^+^ treatments are shown in Figure 7B. The expression of eight specific K^+^ transporters was upregulated at K^+^ deficiency conditions in *P. acinosa* roots. Among them, the expression of *PaHAK5*, *PaKT2*, *PaAKT1*, and *PaHKT1* was strongly induced by K^+^ deficiency treatment. When under K^+^ deficiency, higher expression of these four transporters in roots was activated and they may cooperatively mediate K^+^ uptake from soil in *P. acinosa*. *PaHAK5* and *PaKUP7* showed high expression levels under K^+^ deficiency treatment in leaves and may be the main factors involved in K^+^ transport in leaves. In stems, K^+^ deficiency could greatly induce the expression of *PaAKT2*. *PaAKT2*, *PaHKT1*, and *PaKUP7* appear to be important transporters for mediating K^+^ transport in stems because their basic expression was at high levels in cells of *P. acinosa* stems (Figure 7B).

## 3. Discussion

Potassium distribution is not uniform among the different soil types in different regions. In China, the soil considered to be potassium^-^deficient covers more than 23 million ha. Eighty percent of the Hunan Province area shows potassium deficiency, and half of all farmland shows severe potassium deficiency (available potassium < 50 mg/kg [31]). Interestingly, our survey found that farmers in West Hunan region usually collect and compost *P. acinosa* to fertilize crops. Moreover, *P. acinosa* showed a strong ability to adapt to a low potassium environment and a high capability for potassium accumulation in aerial parts. *P. acinosa* could complete its lifecycle under low K^+^ and did not show an inhibited growth phenotype and leaf chlorosis. Through its growth and development under different K^+^ concentrations, *P. acinosa* demonstrated a strong tolerance to low potassium stress and adaptability to a wide range of external K^+^ levels. 

Land soil potassium content usually ranges from 1% to 2%, and only 2%–10% of the soil potassium is available for direct utilization by plants because most of the potassium is incorporated in the crystal lattice structure of minerals [40,41]. Cellular K^+^ concentrations maintained at 100–150 mM are crucial to plant growth and development. In order to adapt to low available potassium content in soil, plants have developed multiple K^+^ channels, transporters, and other non-specific cation channels, which cooperatively mediate K^+^ import and export in plant cells [42]. Among them, manners driven by thermodynamic and decided by affinity dominate the K^+^ influx across the plasma membrane of root cells against its electrochemical gradient at rhizosphere concentrations less than about 1 mM K^+^ [8,14]. The main K^+^ channel involved in K^+^ nutrition of *Arabidopsis* is an inward-rectifier Shaker K^+^ channel AtAKT1 [25,26,43,44,45,46]. Low-affinity K^+^ uptake could be mediated by AtAKT1, whose gene is expressed mainly in root epidermal cells [47]. The expression of AKT1 did not respond to the external K^+^ [47,48], but K^+^ withdrawal from the growth solution increased the expression of *TaAKT1* from *Triticum aestivum* [49]. AtKC1 appears to be a regulatory subunit for AtAKT1 in root hairs [19,48]. KUP/HAK/KTs are identified to be high-affinity K^+^ transporters and play a role in the homeostasis of K^+^ in cells [50,51,52,53,54,55]. *AtHAK5* was expressed in *Arabidopsis* roots, and it regulated most of the K^+^ uptake in *Arabidopsis* roots [52], and the high expression of the *AtHAK5* gene could be induced by K^+^ deficiency and low K^+^ levels in *Arabidopsis* roots to adapt to the low K^+^ environment [23]. Under the conditions of low K^+^, *hak*5 exhibited a phenotype of sensitivity to low K^+^, with inhibited root growth and a lower K^+^ absorption rate [26]. *AtKUP1*, *AtKUP4*, and *AtHAK5* were identified as key genes for K^+^ uptake in *Arabidopsis* [23,40,56]. Another family of high-affinity K^+^ transporters (HKTs) mainly mediates K^+^/Na^+^ transport and plays an important role in K^+^/Na^+^ homeostasis and salt stress tolerance [29,57,58,59,60,61,62,63,64,65,66]. In addition, almost all monocotyledons have subfamily I and II members of HKTs, and most dicotyledons have one HKT [32]. In this study, 18 members of HAK/KT/KUP, 10 members of AKT, and 1 member of HKT were identified in *P. acinosa* with global transcriptome. These transporters might coordinatively mediate K^+^ uptake and transport in *P. acinosa* and result in high potassium accumulation in plant tissues.

Compared to some other plants, *P. acinosa* shows higher affinity for K^+^ and K^+^ uptake rate [37,38]. *P. acinosa* has acquired certain characteristics regarding K^+^ uptake and accumulation through evolution and natural selection. PaHAK1, a member of the high-affinity K^+^ transporter family in *P. acinosa* was found to mediate K^+^ uptake in roots [32]. Moreover, other HAKs were found to play vitally important roles in K^+^ absorption and transportation. Among them, *PaKT4, PaHAK5, PaKUP6*, *PaKUP7,* and *PaAKT2* were specifically expressed in roots, and the expression levels were much higher than those in stems and leaves. These four genes might be involved in the high-affinity uptake of potassium. The expression of *PaKT2* in leaves was much higher than that in roots and stems. This indicated that *PaKT2* might be mainly involved in the transport of potassium. The expression of *PaAKT1* was not significantly different in different tissues, and thus, it was speculated that *PaKT1* is involved in the uptake and transport of potassium. The expression of *PaHKT1* in stems was much higher than that in roots and leaves. Hence, PaHKT1 might be mainly involved in K^+^ transport.

**Figure 8:**
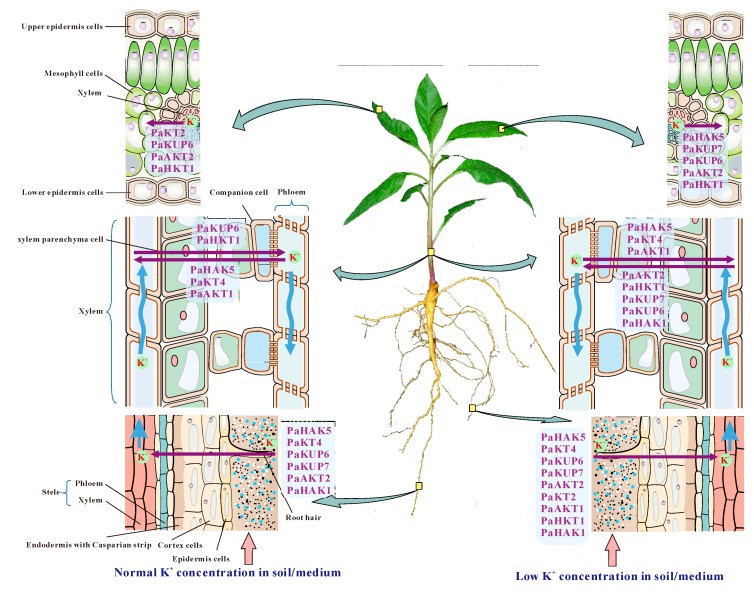
Model of K^+^ uptake/transport mediated by high-affinity K^+^ transporters.

Taken together, we hypothesized the model of K^+^ uptake/transport mediated by high-affinity K^+^ transporters in *P. acinosa* (Figure 8). At normal K^+^ levels, PaHAK5, PaKT4, PaKUP6/7, PaAKT2, and PaHAK1 might mediate K^+^ uptake/transport along the root hair, epidermis cells, and cortex cells, and then transport K^+^ into the stele through symplasts and transmembrane pathways in roots. At K^+^ deficiency, more high-affinity transporters are involved in K^+^ uptake and enhance the K^+^ absorptive capacity of roots because of extremely significant upregulation of *PaHAK5*, *PaKT2*, *PaAKT1*, and *PaHKT1*. Upward K^+^ transport is driven by transpiration flow along the xylem. At the normal K^+^ levels, PaHAK5, PaKT4, PaKUP6, PaAKT1, PaHKT1, and PaHAK1 share responsibility for K^+^ loading and unloading in xylem and maintain K^+^ homeostasis. However, the downregulation of *PaHAK5*, *PaKT4*, and *PaAKT1* at K^+^ deficiency indicate that these three transporters might function in K^+^ loading into stem xylem. Three upregulated transporters (PaAKT2, PaHKT1, and PaKUP7) might be involved in K^+^ unloading from stem xylem to ensure relatively high K^+^ content in the stem. Moreover, highly expressed *PaKT2* and *PaKUP6* and moderately expressed *PaAKT1* and *PaHKT1* mediated the K^+^ transport in leaves at normal K^+^ levels. Under K^+^ deficiency conditions, significant upregulation of *PaHAK5* and *PaKUP7* promoted K^+^ transport in leaves.

In addition, multiple subfamilies of ABC transporters are involved in K^+^ transportation. The ABCB subfamily is widely distributed in eukaryotic cells and has a variety of functions, which are closely related to multidrug resistance, antigen synthesis, pumping peptides and pheromones out of mitochondria, and heavy metal tolerance [67,68,69]. In addition, the ABCB subfamily is involved in the transport of secondary metabolites and other substances [70,71]. The ABCC subfamily is located on vacuole membranes. A series of studies has shown that the ABCC subfamily plays an important role in improving the tolerance of plants and fungi to heavy metals [72,73]. The ABCF subfamily also plays a conservative and important role in cell life activity. It is an important subfamily involved in ribosomal synthesis, transcriptional regulation, and mRNA transfer, and it is also a translation extension factor [74]. Previous studies have provided new evidence that the ABCG subfamily is involved in plant response to biotic and abiotic stresses [75]. ABCG25 and ABCG40 were reported to mediate ABA efflux and influx as part of the plant drought resistance response, respectively [76,77]. In this study, we found that the expression of nearly 30 members of ABC transporters belonging to subfamilies of ABCB, ABCC, ABCF, and ABCG were strongly induced by K^+^ starvation treatment (Figure 4 and Figure 7A). Therefore, we hypothesized that ABC transporters may be important for K^+^ uptake and transport of *P. acinosa* under the condition of K^+^ starvation. Further research is still urgently needed to fully characterize the function of ABC transporters in K^+^ uptake/transport. 

## 4. Conclusions

We collected multiple samples of *P. acinosa* from different regions of Hunan to determine their potassium accumulation characteristics. The results indicated that stems and leaves were the main tissues for high potassium accumulation in *P. acinosa*. We cultured seedlings of *P. acinosa* in solutions of varying K^+^ concentrations, analyzed the potassium contents in different tissues, and found that *P. acinosa* showed a strong ability for K^+^ absorption in roots and a large capability of potassium accumulation in shoots. *P. acinosa* demonstrated good growth and could complete its lifecycle even in severe potassium deficiency soil, showing adaptability under a wide range of external potassium levels. To reveal the mechanism of K^+^ uptake and transport in the high K^+^ plant *P. acinosa*, we compared the expression of K^+^ uptake/transport-related genes at the level of the global transcriptome between K^+^ starved plants and normal cultured plants. Eighteen members of HAK/KT/KUP, 10 members of AKT, and 1 HKT were identified in *P. acinosa* with global transcriptome analysis. These transporters might coordinatively mediate K^+^ uptake and transport in *P. acinosa* and result in high potassium accumulation in plant tissues. The model of K^+^ uptake/transport mediated by high-affinity K^+^ transporters was hypothesized in different tissues of *P. acinosa*. In addition, we found that expression of nearly 30 members of ABC transporters, belonging to subfamilies of ABCB, ABCC, ABCF, and ABCG, was strongly induced by K^+^ starvation treatment. This indicated that ABC transporters may be important for K^+^ uptake and transport of *P. acinosa* under conditions of K^+^ deficiency.

## 5. Materials and Methods

### 5.1. Plant Collection from Fields

*P. acinosa* plants were collected from fields in Hunan. All samples were brought into the laboratory, then washed thoroughly with tap water and rinsed with deionized water. The samples were divided into roots, stems, and leaves, then blotted and dried at 105 °C for 30 min, then at 80 °C until their weights remained constant. The dried samples were used for the measurement of K^+^ content.

### 5.2. Plant Materials and Growth Conditions

The seeds of *P. acinosa* were soaked in concentrated sulfuric acid for 10 min to carbonize the seed shell and then washed 5 times using distilled water. The seeds were evenly sprinkled in sand-filled culture pots and covered with plastic wrap to maintain the temperature and humidity. Culture pots were placed in a greenhouse at 22 °C under 16 h light followed by 8 h dark. The plastic wrap was removed 5 days later, and the seedlings were cultured under the same conditions. In this culture procedure, the sand in pots were irrigated with Hoagland solution.

### 5.3. K^+^ Treatment for Physiological Tests

The 20-day-old *P. acinosa* seedlings were transplanted to pots with 10 kg sand (no applicable nutrition) and then irrigated with 2 L modified Hoagland solution (PH = 5.8) containing 1 mM NH_4_H_2_PO_4_, 3 mM Ca(NO_3_)_2_, 2 mM MgSO_4_·7H_2_O, and 0.65 mM CaCl_2_, plus micronutrients and Fe–NaEDTA. The 0.5, 3.0, and 12 mmol/L K^+^ treatments were supplemented by respectively adding 0.5, 3, and 12 mM KCl. In addition, the three K^+^ treatments were supplemented with 1.5 mM NH_4_NO_3_. There were 10 pots for each treatment and 3 plants in each pot. Plants were cultured in a greenhouse at 22 °C under 16 h light followed by 8 h dark.

The 60-day-old plants at vegetative growth stage were measured for plant height and leaf area, and 120-day-old plants at reproductive growth stage were measured for plant height, leaf area, chlorophyll content, and photosynthetic rate. The number and length of infructescences were measured after berry ripening. The seeds were harvested and dried, and then the 1000-seed weight was determined. 

### 5.4. K^+^ Treatment for Transcriptome Sequencing

Twenty-day-old *P. acinosa* seedlings were transferred into modified Hoagland solution (PH = 5.8) containing 1 mM NH_4_H_2_PO_4_, 3 mM Ca(NO_3_)_2_, 2 mM MgSO_4_·7H_2_O, and 0.65 mM CaCl_2_, plus micronutrients and Fe–NaEDTA. The 0 and 3.0 mmol/L K^+^ treatments refer to supplementation with either 0 or 3 mM KCl. In addition, two K^+^ treatments involved supplementation with 1.5 mM NH_4_NO_3_. Seedlings cultured in the greenhouse at 22 °C under light and air was constantly supplemented into modified Hoagland solution through an air pump. Six hours later, the roots from five identical seedlings of each treatment were collected as one sample. Three independent samples in each treatment were used to extract total RNA and then applied in transcriptome sequencing to screen gene expression. 

### 5.5. Measurement of Chlorophyll Content and Photosynthetic Rate in Leaves

The eighth leaves were collected from three treatments in each pot and the veins were taken out. Then, 0.3 g of cut-up samples from each pot were immersed in 20 mL of 80% acetone in test tubes. The test tubes were placed in darkness for 12 h and rotated several times during this period until all samples became white in color. The solution was filtered with a funnel. The test tube and funnel were washed with distilled water 3 times, and the washing solution was transferred to a 200 mL volume bottle and adjusted to 200 mL. The absorbance value of each acetone–chlorophyll solution was detected at 663 and 645 nm using a T6 spectrophotometer (Pgeneral, Beijing, China). Chlorophyll content was calculated according to the Arnon equation: chlorophyll content (mg/L) = 20.2 × A_645_ + 8.02 × A_663_ [78].

Photosynthetic rate was measured on 15 pieces of mature leaves for each treatment using a portable photosynthesis analyzer (LI-6400XT, Li-COR, Lincoln, Nebraska, USA) under conditions of saturating light and water availability. During the measurement processes, plants were kept under conditions of high soil water availability. The temperature in the leaf chamber was maintained between 25 and 33 °C, and all measurements were carried out between 8:00 AM and 11:00 AM.

### 5.6. K^+^ Content Analysis through ICP–MS

The dry samples (0.1 g) were digested in HNO_3_/H_2_O_2_ (7:1.5, *v/v*) at 120 °C for 10 min, then kept at 139 °C for 20 min. The samples were taken out and put into an Acid Removing Device (Yiyao Instrument, Beijing, China) at 120 °C for 30 min to remove the remaining HNO_3_/H_2_O_2_ in solution. The digested solution was cooled to below 50 °C and dissolved in 100 mL deionized water. A 2.5 mL aliquot was taken to measure the potassium content by ICP–MS (Agilent 7900, Santa Clara, California, USA). The general settings of the 7900 ICP–MS are listed in Table 2. K^+^ Translocation Coefficients = K^+^ content in shoots/ K^+^ content in roots.

### 5.7. Transcriptome Analysis

Total RNA was extracted from tissue samples using Plant RNA Purification Reagent (Invitrogen, Carlsbad, California, USA), and then the concentration and purity of RNA were determined by Nanodrop 2000 (Thermo, Waltham, Massachusetts, USA) and agarose gel electrophoresis. A library was constructed by an Illumina TruSeq^TM^ RNA Sample Prep Kit, and RNA sequencing was conducted using an IlluminaHiSeq 4000 SBS Kit v3-HS (Illumina, Santiago, California, USA) for 200 cycles. Raw reads were processed to obtain clean reads by removing low quality bases at the 3’ end and the adapter sequences. Transcriptome de novo assembly was carried out using Trinity software. Trinity software was used to assemble the transcriptome de novo and predict open reading frames (ORFs) of the unigenes. The longest ORFs of the putative genes were annotated against the non-redundant (NR), String, SwissProt, and KEGG databases using the BLAST algorithm with the typical cutoff E-value < 10^−5^. The GO (Gene Ontology) function, COG (Clusters of Orthologous Groups of proteins), and KEGG (Kyoto Encyclopedia of Genes and Genomes) pathway enrichment analyses were performed using Blast2GO software (Bio Bam Bioinformatics SL, Valencia, Spain).

### 5.8. Real-Time Quantitative PCR Analysis

The 20-day-old seedlings were transferred into modified Hoagland solution (pH = 5.8) with 0.5 mmol/L and 3.0 mmol/L K^+^ and cultured at 22 °C under light. Air was constantly supplemented into modified Hoagland solution through an air pump. 6 h later, tissues (root, stem, or leaf) from 5 individual plants of each treatment were collected respectively for analysis of gene expression in different tissues through real-time quantitative PCR (RT-qPCR). Primers based on the cDNA sequences of transcriptome sequences are listed in Table 3. Total RNA was purified from gDNA contamination and reverse-transcribed to cDNA using TransScript One-Step gDNA Removal and cDNA Synthesis SuperMix (Transgen, Beijing, China). A PCR program was performed on a CFX96-CFX384 Real Time PCR System (Bio-Rad, Hercules, California, USA) in a final volume of 20 μL containing 1 μL of a 1/5 diluted cDNA template, 10 μL of the 2×ChamQ SYBR Master Mix (Vazyme, Nanjing, China), and 0.5 μL (10 μM) of forward and reverse primers. Thermal cycler settings consisted of an initial hold at 95 °C for 30 s, then 45 cycles of 95 °C for 10 s, and 60 °C for 30 s. Three biological replicates were analyzed (*n* = 3). The actin gene of *P. acinosa* was used as internal standard.

### 5.9. Gene Searching and Phylogenetic Analysis

The sequences of target genes were downloaded from NCBI. The phylogenetic tree was constructed using neighbor-joining in MEGA6.0 [79,80].

### 5.10. Statistical Analysis

All experiments were conducted in at least three biological replicates. Statistical significance was evaluated using Graph Pad Prism5 software. 

## Figures and Tables

**Table 1:** Basic data of RNA sequencing in the samples of TP01 and TP02.

RawData	TotalReads	TotalBases	Error%	Q20%	Q30%	GC%
TP01-1	34,165,110	4,881,892,216	0.0134	97.5	91.74	51.45
TP01-2	33,169,330	4,878,900,814	0.0138	97.3	91.73	51.40
TP01-3	35,157,926	4,881,996,121	0.0136	97.4	91.78	51.47
TP02-1	75,835,214	10,521,500,724	0.0103	98.7	95.40	47.4
TP02-2	75,831,068	10,516,553,023	0.0101	98.2	95.27	46.9
TP02-3	75,834,548	10,520,436,458	0.0102	98.6	95.35	47.3

**Table 2:** 7900 ICP–MS operating parameters.

Parameter	Value	Parameter	Value
Radio frequency power	1.45 V	Orifice of sampling cone	1.0 mm
Carrier gas flow rate	0.85 L/min	Orifice of skimmer cone	0.4 mm
Mixed gas flow rate	0.28 L/min	Sample depth	7.0 mm
Plasma gas flow rate	15.0 L/min	Sample uptake flow rate	0.1 r/s
Auxiliary gas flow rate	1.0 L/min	Sample uptake amount	0.4 mL/min
Helium flow rate	5 mL/min	Spray chamber temperature	2 °C

**Table 3:** Primers used in this study.

Seq_ID	Gene Name	Primer(5’-3’)
c126889g1_i1	*PaHAK5*	P1 ATGGATCTCAGCAGGATGGG
		P2 TTCCATTGTCGTTTGCCCTG
c219737g1_i1	*PaKT2*	P1 GAGCCTTCTGCCAAAC
		P2 CCATCTCCAACCACCA
c56511g1_i1	*PaKT4*	P1 GGTCGGTGTACGAGGAT
		P2 TGAGGCTAATGTGAGGAA
c123766g1_i1	*PaKUP6*	P1 CGGGACCCTCAAGAAG
		P2 GCCTATGCCACGGACT
c125123g6_i1	*PaKUP7*	P1 TCTGAGGCTATGTTTGCCGA
		P2 GTAGCTGTCGTCATTGCTCG
c108651g2_i1	*PaAKT1*	P1 TTCAGAAGGGTTGCAACAGC
		P2 AAATTCCACGGCTCCAGAGA
c121941g1_i1	*PaAKT2*	P1 GGAGCAGGGAAATGTTGTGG
		P2 TAAGGCTGAGGGATGTTGCA
c72107g1_i1	*PaHKT1*	P1 CGGTTCTTGTGCTGTT
		P2 TGCTTCTTGTCGCTGA
Actin		P1 CTTGACTTTGAGCAGGAATCGGAGA
		P2 ACCTGCTGCTTCCATACCTATCAAT

## Supplementary Materials

The following are available online at https://www.mdpi.com/2223-7747/9/4/470/s1, Table S1: Potassium content in different species of plants.
