# Supplementary material for: Multiple High-Affinity K+ Transporters and ABC Transporters Involved in K+ Uptake/Transport in the Potassium-Hyperaccumulator Plant Phytolacca acinosa Roxb
Source: Plants (Basel). 2020 Apr 8;9(4):470. doi: 10.3390/plants9040470 (PMC7238005; doi:10.3390/plants9040470)
Supplement: Supplementary file 1 [file plants-09-00470-s001.docx]

Table S1. Potassium content in different species of plants.

| Species | K^+^ content (%) in dry matter | Reference |
| --- | --- | --- |
| *Oryza sativa,* early var | 2.90-4.02 | Hu, D.J. *et al.* (1980) |
| *Oryza sativa,* seconed var | 1.78-2.40 |  |
| *Alternanthera philoxeroides* | 5.88-11.69 |  |
| *Hydrocharis dubia* | 6.00-7.75 |  |
| *Vallisneria spiralis* | 6.20-8.08 |  |
| *Lemna pareiostuta* | 4.40-6.10 |  |
| *Callitricho stagnalis* | 3.15-3.55 |  |
| *Patamogeton crispus* | 4.28-5.65 |  |
| *Ceratophyllum demersum* | 6.00 |  |
| *Potamogeton distinctus* | 3.42 |  |
| *Hydrilla verticillata* | 4.42 |  |
| *Trapa maximouyczee* | 1.61 |  |
| *Salvinia natans* | 2.50 |  |
| *Eleocharis yokoscensis* | 5.10 |  |
| *Azolla imbricata* | 4.01 |  |
| *Chara fragilis* Desv. | 3.16 |  |
| *Chara fragilis* Desv.Var. | 5.93 |  |
| *Robinia pseudoacacia* | 3.10 |  |
| *Medicago sativa* | 3.30-4.05 |  |
| *Melilotus albus* | 3.00-3.57 |  |
| *Melilotus suaveolens* Ledeb.Var. | 2.20-2.85 |  |
| *Astragalus sinicus* | 3.60-4.50 |  |
| *Astragalus sinicus*.Var | 0.91-2.90 |  |
| *Pisum sativum* L. | 3.20 |  |
| *Pisum sativum* L.Var | 0.87-4.18 |  |
| *Avena fatua* | 2.60-2.92 |  |
| *Helianthus tuberosus* | 4.40-5.10 |  |
| *Lycopersicon esculentum* | 4.10 | Besford, R.T. *et al.* (1975) |
| *Helianthus anmuus* | 10.00 | Spear, S.N. *et al.* (1978) |
| *Zea mays* | 2.00 | Tyber, E.H. *et al.* (1946) |
| *Paspalum notatum* | 3.00-3.40 | Gammon, N.J.R. *et al.* (1952) |
| *Cynodon dactylon* | 3.00-3.10 |  |
| *Trifolium repens* | 5.00 |  |
| *Nicotiana tabacum* (100 kg/ha K_2_O) | 6.63-6.76 | Tari, M. *et al.* (2018) |
| *Nicotiana tabacum* (200 kg/ha K_2_O) | 6.82-6.86 |  |
| *Hordeum vulgare* (80 kg/ha K fertilization) | 1.80 | Zörb, C. *et al*. (2014) |
| *Hordeum vulgare* (160 kg/ha K fertilization) | 1.90 |  |
| *Triticum aestivum* (80 kg/ha K fertilization) | 1.65 |  |
| *Triticum aestivum* (100 kg/ha K fertilization) | 1.70 |  |

Hu, D.J. Yang, M.Y.; Liu, G.H. Studies on high potassium plants. *Journal of Hunan Agricultural University (Natural Sciences)* **1980**, 4, 8-16.

Besford, R.T. [Effect of potassium nutrition on leaf protein concentrations and growth of young tomato plants](http://www.onacademic.com/detail/journal_1000034284833410_245f.html). *Plant and Soil* **1975**, 42, 441-451.

Spear, S.N.; Asher, C.J.; Edwards, D.G. Response of cassava, sunflower, and maize to potassium concentration in solution I. Growth and plant potassium concentration. *Field Crops Research* **1978**, 1, 347-361.

Tyber, E.H. The relation of corn yields to leaf nitrogen, phosphorus and potassium content. *Soil Science Society of America, Proceedings* **1946**, 11, 317-323.

Gammon, N.J.R.; Blue, W.G. Potassium requirements for pastures. Proceedings of the Soil Science Society of Florida **1952**, 12, 154-156.

Tari, M.; Afzal, M.N.; Muhammad, D.; Ahmad, S.; Shahzad, A.N.; Kiran, A.; Wakeel, A. Relationship of tissue potassium content with yield and fiber quality components of Bt cotton as influenced by potassium application methods. *Field Crops Research* **2018**, 229, 37-43.

Zörb, C.; Senbayram, M.; Peiter, E. Potassium in agriculture-status and perspectives. *Journal of Plant Physiology*, **2014**, 171, 656-669.
